# Adoption of a Portal for the Primary Care Management of Pediatric Asthma: A Mixed-Methods Implementation Study

**DOI:** 10.2196/jmir.5610

**Published:** 2016-06-29

**Authors:** Alexander G Fiks, Nathalie DuRivage, Stephanie L Mayne, Stacia Finch, Michelle E Ross, Kelli Giacomini, Andrew Suh, Banita McCarn, Elias Brandt, Dean Karavite, Elizabeth W Staton, Laura P Shone, Valerie McGoldrick, Kathleen Noonan, Dorothy Miller, Christoph U Lehmann, Wilson D Pace, Robert W Grundmeier

**Affiliations:** ^1^ The Children's Hospital of Philadelphia Philadelphia, PA United States; ^2^ The University of Pennsylvania Philadelphia, PA United States; ^3^ The American Academy of Pediatrics Elk Grove Village, IL United States; ^4^ University of Colorado Denver Denver, CO United States; ^5^ The American Academy of Family Physicians Leawood, KS United States; ^6^ Vanderbilt University Nashville, TN United States

**Keywords:** asthma, electronic health records, health information technology

## Abstract

**Background:**

Patient portals may improve communication between families of children with asthma and their primary care providers and improve outcomes. However, the feasibility of using portals to collect patient-reported outcomes from families and the barriers and facilitators of portal implementation across diverse pediatric primary care settings have not been established.

**Objective:**

We evaluated the feasibility of using a patient portal for pediatric asthma in primary care, its impact on management, and barriers and facilitators of implementation success.

**Methods:**

We conducted a mixed-methods implementation study in 20 practices (11 states). Using the portal, parents of children with asthma aged 6-12 years completed monthly surveys to communicate treatment concerns, treatment goals, symptom control, medication use, and side effects. We used logistic regression to evaluate the association of portal use with child characteristics and changes to asthma management. Ten clinician focus groups and 22 semistructured parent interviews explored barriers and facilitators of use in the context of an evidence-based implementation framework.

**Results:**

We invited 9133 families to enroll and 237 (2.59%) used the portal (range by practice, 0.6%-13.6%). Children of parents or guardians who used the portal were significantly more likely than nonusers to be aged 6-9 years (vs 10-12, *P*=.02), have mild or moderate/severe persistent asthma (*P*=.009 and *P*=.04), have a prescription of a controller medication (*P*<.001), and have private insurance (*P*=.002). Portal users with uncontrolled asthma had significantly more medication changes and primary care asthma visits after using the portal relative to the year earlier (increases of 14% and 16%, respectively). Qualitative results revealed the importance of practice organization (coordinated workflows) as well as family (asthma severity) and innovation (facilitated communication and ease of use) characteristics for implementation success.

**Conclusions:**

Although use was associated with higher treatment engagement, our results suggest that achieving widespread portal adoption is unlikely in the short term. Implementation efforts should include workflow redesign and prioritize enrollment of symptomatic children.

**ClinicalTrial:**

Clinicaltrials.gov NCT01966068; https://clinicaltrials.gov/ct2/show/NCT01966068 (Archived by WebCite at http://www.webcitation.org/6i9iSQkm3)

## Introduction

Patient portals, Web-based health care applications that enable patients to interact and communicate with their health care providers from outside the office [1], offer a resource to improve communication between patients and clinicians between visits. Patient portal use has increased recently [2]; however, adoption has not been rapid [3], and overall rates of sustained use remain low [4]. Recent research suggests that to effectively engage patients as portal users, several barriers may need to be overcome. For organizations, leadership challenges, marketing problems, and limited staff commitment have constrained portal adoption [5]. Studies have also found that patients who are white and have more health problems are more likely to use portals than others [4,6-8]. In addition, portals have not been as widely used in pediatrics as in the adult setting.

Pediatric asthma is an ideal condition for evaluating the feasibility of implementing portals to facilitate the management of chronic disease in practice. More than 7 million children in the United States have asthma [9], the most common pediatric chronic illness. Asthma is associated with lower quality of life [10,11], more missed days of school for children and work for parents [11-13], higher rates of hospitalization, emergency department visits [14], and death [15]. As appointment follow-up varies in pediatric primary care [16,17], and time constraints of office visits limit discussion, portals may facilitate decision making between families at home and primary care practices. However, the feasibility of using portals to collect patient-generated health information and portals’ impact on clinical care across diverse pediatric settings has not been established.

This study evaluated the determinants of implementation success for a portal in pediatric primary care to facilitate communication between families and clinicians regarding treatment concerns and goals, asthma symptoms, medication use, and side effects. In a subset of children with poorly controlled asthma, we further assessed the impact of portal use on asthma management, as clinical impact justifies implementation efforts. Finally, we qualitatively evaluated barriers to and facilitators of portal use experienced by families and primary care practices.

## Methods

### Setting

Twenty primary care practices were enrolled from 2 practice-based research networks: Pediatric Research in Office Settings (PROS) of the American Academy of Pediatrics and the Pediatric Research Consortium (PeRC) of the Children’s Hospital of Philadelphia (CHOP). PeRC is a hospital-owned primary care practice-based research network with 31 primary care practices and 231 clinicians in Pennsylvania and New Jersey [18]; PROS includes 728 practices and 1831 clinicians across the United States and Canada. A convenience sample of 9 PROS and 11 PeRC practices was enrolled.

### Study Population

Eligible participants included English-speaking parents or guardians (subsequently “parents”) of children aged 6-12 years, who received treatment at a participating practice, had an asthma diagnosis at the time of recruitment, and had an office visit during the past 12 months.

### Recruitment

To ensure that low-income children were represented, study practices were required to have ≥20% of children insured by Medicaid or the Children’s Health Insurance Program. Each practice was contacted by the investigators (AF, SF), invited to participate, and received an in-person or remote presentation of study procedures. After enrollment, rosters of all eligible children were generated from the electronic health record (EHR) to identify families for recruitment. Because of technical differences in the portals between PeRC and PROS, we tailored recruitment by setting. In PeRC, the study team mailed up to 2 letters to all eligible families, inviting them to call the study team to enroll. Parents provided verbal consent over the phone and enrolled in the MyAsthma portal. In PROS, families were mailed letters with a link to the portal website where families could enroll and consent. Telephone recruitment was used for a random sample of 50 families at each practice who did not respond to the letters. These phone calls were completed by the study team in PeRC and by the primary care practice clinicians/staff in PROS. Informational cards were available and posters were on display in participating offices. Enrolled parents received a $10 incentive for using the portal. PROS practices received $1000 for participating in the study, recognizing the additional work required for data extraction from independent practices.

### The MyAsthma Portal

**Figure 1:**
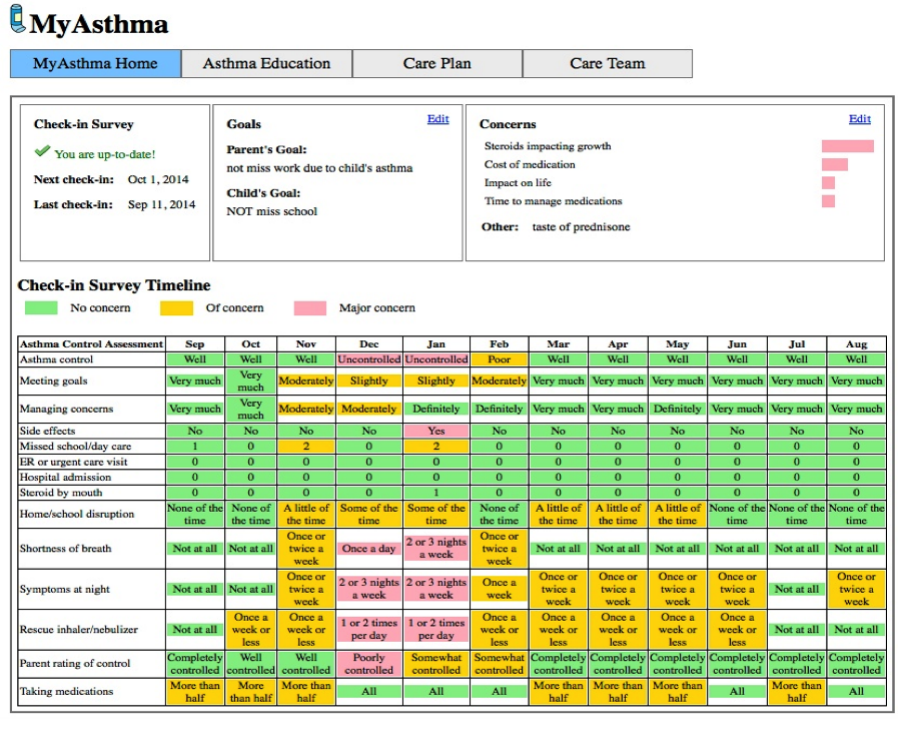
The MyAsthma Portal-PeRC Practices. In PeRC, MyAsthma was embedded in an existing patient portal (MyChart, Epic, Verona, WI, USA) already implemented by The Children’s Hospital of Philadelphia. ©2014 The Children’s Hospital of Philadelphia. All Rights Reserved.

**Figure 2:**
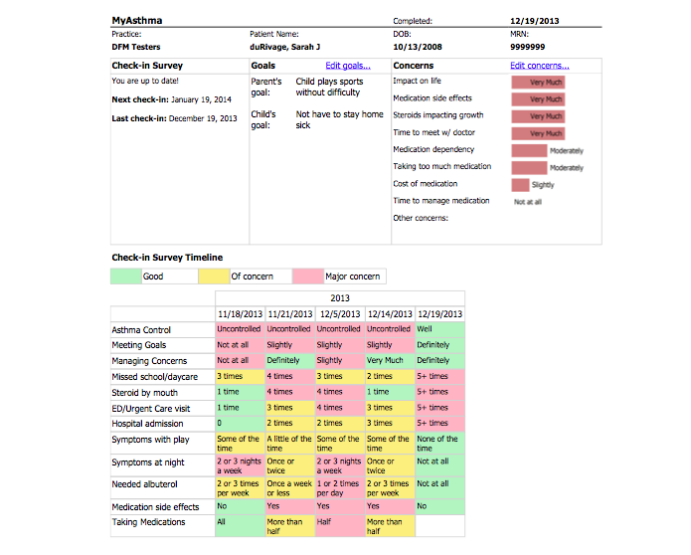
The MyAsthma Portal-PROS Practices. In PROS, MyAsthma was available to families through Integrated Health Connect (IHealth Connect), a website developed by the University of Colorado. A test patient is shown.

The MyAsthma portal was developed and tested at CHOP to facilitate shared decision making and improve asthma outcomes [19,20]. The portal was designed through a user-centered process including interviews and focus groups with 7 parents of children with asthma and 51 clinical team members, including doctors, nurse practitioners, and nurses. Functions of the portal were designed to reflect features families and clinicians prioritized, and iterative usability testing with parents and clinicians refined the portal system [19]. MyAsthma provides educational material; enables sharing of families’ treatment concerns, goals, asthma symptoms, medication adherence, and side effects with the primary care clinical team; tracks asthma control over time for families through the portal and clinicians through the EHR; and provides decision support to both families and clinicians regarding asthma control and side effects. On enrollment, families entered information about their treatment concerns and goals and completed an asthma control survey that assessed symptoms, medication adherence, and side effects. We used a version of the Asthma Control Test [21] that had been modified slightly to allow for parent proxy report of child symptoms. Subsequently, families were prompted by email each month to complete the asthma control survey. At CHOP, MyAsthma is embedded within an existing patient portal (MyChart, Epic, Verona, WI, USA; Figure 1). In PROS, families interacted with the portal through a Web interface (Figure 2), and decision support was provided on screen to families and via fax to practices based on asthma control survey results.

### Outcomes

**Figure 3:**
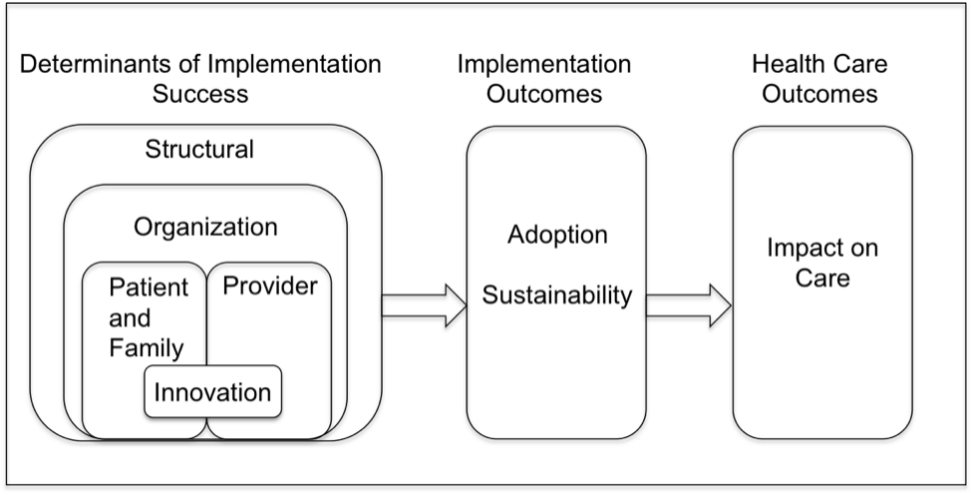
Conceptual model of factors affecting the implementation of health innovations, adapted from [22].

The primary outcomes included adoption (completion of at least one portal survey during the study period) and sustained use (completion of at least two surveys) of MyAsthma; outcomes were informed by an evidence-based conceptual model of factors influencing implementation success (Figure 3) [22]. We assessed additional outcomes (asthma office visit or asthma medication refill/change within 30 days of survey completion) in a subgroup of children who had uncontrolled asthma according to the results of their first asthma control survey. We focused on these actions because they are appropriate measures to take in response to poor asthma control. These data were extracted from each child’s EHR. In addition, parents and guardians reported whether they were more or less likely to (1) speak to their child’s doctor, (2) make a change to their child’s medication dosage, or (3) make a change to their home environment after using the portal using a 5-point Likert scale.

### Covariates

We extracted the following covariates from the EHR: patient age, sex, race and ethnicity, asthma severity (mild intermittent, mild persistent, moderate or severe persistent), insurance status (public vs private), and asthma controller medication use at study start (including inhaled steroids, montelukast, combination of inhaled steroid or long-acting β-agonists, and oral steroids). Parent-level covariates were collected via survey from enrolled participants and included age, race and ethnicity, educational attainment, employment status, and relationship to the child. Practice-level covariates included urbanicity (rural, suburban, or urban) and US census region (Northeast, South, Midwest, West).

### Statistical Analysis

The study population was described using proportions, means, and standard deviations. Characteristics of children whose parents/guardians completed the portal survey were compared with those of children whose parents and/or guardians did not, using chi-square and *t* tests. Fisher exact tests were used for categorical data with sparse cell counts, and Mann–Whitney *U* tests were used for skewed continuous variables. Characteristics of children with sustained use were compared with those of children whose parents or guardians only completed the portal survey once. Multivariable logistic regression was used to model the association of patient characteristics and practice site with portal adoption to identify factors associated with adoption. The proportion of families who enrolled in the portal in response to a mailed letter versus a telephone call was also compared descriptively.

In the subgroup of patients with uncontrolled asthma, we described the proportion of children with an asthma office visit or medication refill or change within 30 days of survey completion. In a sensitivity analysis, we repeated these analyses with a period of 14 days. Furthermore, for each child, we compared these results to the same 1-month period a year earlier to assess whether rates of office visits and medication adjustments changed. We calculated 95% CIs around the change in proportions between years using logistic regression with the margins command in Stata (StataCorp, College Station, TX, USA). We also described parent responses to survey questions regarding the impact of portal use.

All analyses were completed using Stata, version 13.1. The Institutional Review Boards at the American Academy of Pediatrics (reference number: 13 FI 01) and CHOP (reference number: 13-010285) approved this study. All parents provided informed consent and child assent was waived as all information was collected from parents only and because children would not necessarily be readily available when parents were consented by telephone.

### Qualitative Study

To evaluate implementation success and identify barriers to and facilitators of portal adoption (Figure 3) [22], trained research assistants on the study team used an interview guide based on our conceptual model to conduct 22 semistructured interviews by phone with parents, purposively sampled to include enrolled (14) and unenrolled (8) from both PROS (7) and PeRC (15), and 10 focus groups (PeRC in-person, PROS by phone, purposively sampled to include diverse representation from both networks) with 46 clinicians. All interviews were recorded then transcribed and coded using NVivo10 (QSR, Cambridge, MA, USA) and interpreted in the context of the conceptual model. Differences in coding were resolved by team consensus.

## Results

### Adoption and Sustained Use

**Figure 4:**
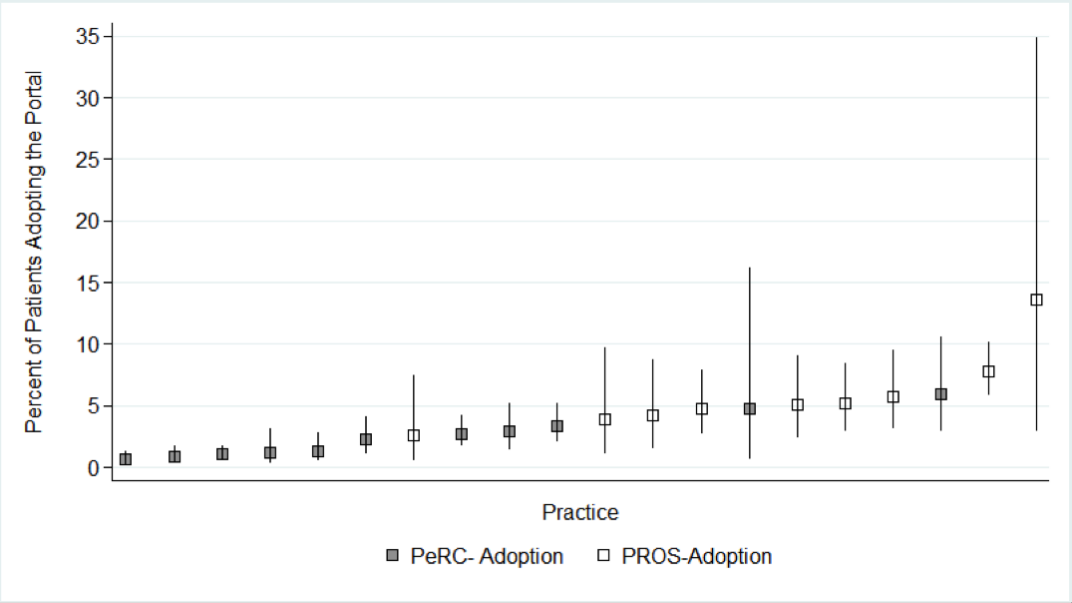
Practice-level variability in portal adoption within 2 pediatric primary care networks. Range 0.6%-13.6%. CIs account for practice size (smaller practices have wider intervals).

Few invited families adopted the portal. Out of 9133 eligible patients, 237 (2.59%) completed the portal asthma control survey at least once (adoption). A total of 156 (65.8 % of portal adopters, 1.71% of eligible parents) completed the portal survey more than once (sustained use). Adoption varied widely across practices (0.6%-13.6%; Figure 4). Similarly, sustained use ranged from 0.0% to 13.6%. Reflecting a high level of quality of care, 93.42% of children at PeRC practices with persistent asthma were on a controller medication at baseline. Data on asthma severity were not available in PROS.

Portal users were more likely to have children aged 6-9 years (*P*=.009), to be white (*P*<.001), to be privately insured (*P*<.001), to have mild persistent or moderate or severe persistent asthma (*P*=.002), to be on an asthma controller medication (*P*<.001), and to be receiving a greater number of asthma medications at baseline on average than those who did not use the portal (*P*<.001; Table 1). In addition, those with persistent asthma were twice as likely to use the portal versus those with intermittent asthma (2.37% vs 1.25% at CHOP practices where these data were available, *P*<.001). Sustained portal users were more likely than one-time users to have children who were Hispanic (*P*=.02), have private insurance (*P*=.02), and be from the Northeast (Table 2, *P*=.001). Parents who had sustained use of the portal also had higher educational levels (*P*=.002).

In multivariable logistic regression, the following characteristics were positively associated with portal adoption: receipt of a controller medication at baseline (odds ratio, OR, 2.0, [95% CI 1.5, 2.7]), private insurance (2.0 [1.3, 3.1]), lower child age (1.4 [1.1, 1.9]), and greater asthma severity (1.9 [1.2, 3.0] mild and 1.9 [1.0, 3.5] for moderate or severe persistent versus intermittent; Table 3).

**Table 1:** Characteristics of families of children with asthma who used the MyAsthma portal compared with families who did not—portal adoption (used portal at least once).

Characteristic at study start			Used portal ≥ once, N (%)	Did not use portal, N (%)	*P* value^a^
**Child characteristics**				
	N Children		237	8896	
	Age, years				
		6-9	175 (73.8)	5844 (65.7)	.009
		10-12	62 (26.2)	3052 (34.3)	
	Male		136 (57.4)	5168 (58.1)	.8
	Race^b^				
		White	144 (61.5)	3110 (35.2)	<.001
		Black/African American	75 (32.1)	4789 (54.1)	
		Asian	4 (1.7)	194 (2.2)	
		Other race	11 (4.7)	753 (8.5)	
	Hispanic ethnicity		10 (4.3)	534 (6.1)	.3
	Public insurance^c^		41 (34)	4025 (58.7)	<.001
	Asthma severity^c^				
		Intermittent	49 (41.2)	3857 (57.2)	.002
		Mild persistent	51 (42.8)	2007 (29.8)	
		Moderate/severe persistent	19 (16.0)	873 (13.0)	
	On asthma controller medication		162 (68.4)	4890 (55.0)	<.001
	Mean number of asthma medications (SD)		1.6 (1.4)	1.1 (1.4)	<.001
**Practice characteristics**					
	Practice Setting				
		Urban	64 (27.0)	4592 (51.6)	<.001
		Rural	52 (21.9)	1309 (14.7)	
		Suburban	121 (51.1)	2995 (33.7)	
	Region				
		Northeast	120 (50.6)	7000 (78.7)	<.001
		South	22 (9.3)	373 (4.2)	
		Midwest	67 (28.3)	960 (10.8)	
		West	28 (11.8)	563 (6.3)	
**Parent characteristics** ^d^					
	N parents completing survey		237		
	Mean parent age (SD)		37.7 (5.8)		
	Relation to child: mother		228 (96.2)		
	Race				
		White	148 (62.4)		
		Black/African American	68 (28.7)		
		Asian	4 (1.7)		
		Other race	17 (7.2)		
	Hispanic ethnicity		17 (7.2)		
	Parent education				
		High school or less	34 (14.3)		
		Some college/associates	81 (34.2)		
		Bachelor’s or higher	122 (51.5)		
	Parent employment status				
		Working outside the home	157 (66.2)		
		Self-employed	13 (5.5)		
		Working without pay	43 (18.1)		
		Unemployed	24 (10.1)		

^a^*P* values calculated using the chi-square test, Fisher exact test, *t* test, and Mann–Whitney *U* test.

^b^Race was missing for 53 children (0.6%), ethnicity was missing for 154 (1.7%).

^c^Data on insurance type and asthma severity were only available for PeRC patients (7120 or 78.0% of the total).

^d^Parent characteristics were only collected from families who enrolled in the study (N=237 that completed at least one survey). As such, we are unable to compare these parents with the overall population.

**Table 2:** Characteristics of families of children with asthma who used the MyAsthma portal compared with families who did not—sustained portal use (used portal more than once).

Characteristic at study start			Used portal more than once, N (%)	Used portal one time only, N (%)	*P* value^a^
**Child characteristics**					
	N Children		156	81	
	Age, years				
		6-9	115 (73.7)	60 (74.1)	.9
		10-12	41 (26.3)	21 (25.9)	
	Male		85 (54.5)	51 (63.0)	.2
	Race^b^				
		White	89 (57.8)	55 (68.8)	.2
		Black/African American	54 (35.1)	21 (26.3)	
		Asian	4 (2.6)	0 (0.0)	
		Other race	7 (4.5)	4 (5.0)	
	Hispanic ethnicity		10 (6.5)	0 (0.0)	.02
	Public insurance^c^		27 (28.7)	14 (53.9)	.02
	Asthma severity^c^				
		Intermittent	37 (39.8)	12 (46.2)	.5
		Mild persistent	39 (41.9)	12 (46.2)	
		Moderate/severe persistent	17 (18.3)	2 (7.7)	
	On asthma controller medication		110 (70.5)	52 (64.2)	.3
	Mean number of asthma medications (SD)		1.5 (1.4)	1.8 (1.5)	.4
**Practice characteristics**					
	Practice Setting				
		Urban	44 (28.2)	20 (24.7)	.2
		Rural	29 (18.6)	23 (28.4)	
		Suburban	83 (53.2)	38 (46.9)	
	Region				
		Northeast	94 (60.3)	26 (32.1)	.001
		South	11 (7.1)	11 (13.6)	
		Midwest	35 (22.4)	32 (39.5)	
		West	16 (10.3)	12 (14.8)	
**Parent characteristics**					
	N Parents completing survey		156	81	
	Mean parent age (SD)		38.1 (5.5)	37.1 (6.5)	.2
	Relation to child: Mother		149 (95.5)	79 (97.5)	.8
	Race				
		White	93 (59.6)	55 (67.9)	.4
		Black/African American	48 (30.8)	20 (29.4)	
		Asian	4 (2.6)	0 (0.0)	
		Other race	11 (7.1)	6 (7.4)	
	Hispanic ethnicity		13 (8.3)	4 (4.9)	.4
	Parent education				
		High school or less	17 (10.9)	17 (21.0)	.002
		Some college/associates	46 (29.5)	35 (43.2)	
		Bachelor’s or higher	93 (59.6)	29 (35.8)	
	Parent employment status				
		Working outside the home	111 (71.2)	46 (56.8)	.06
		Self-employed	5 (3.2)	8 (9.9)	
		Working without pay	25 (16.0)	18 (22.2)	
		Unemployed	15 (9.6)	9 (11.1)	

^a^*P* values calculated using the chi-square test, Fisher exact test, *t* test, and Mann–Whitney *U* test.

^b^Race was missing for 3 children (1.3%), ethnicity was missing for 5 (2.1%).

^c^Data on insurance type and asthma severity were only available for PeRC patients (119 or 50.2% of the total).

**Table 3:** Child characteristics associated with portal adoption in multivariable logistic regression.^a^

Characteristic at study start		Adoption versus no adoption, odds ratio (95% CI)^a^	*P* value
**Child age 6-9 (vs 10-12) years**		1.4 (1.1, 1.9)	.02
**Male sex**		0.9 (0.7, 1.2)	.6
**Race**			
	White	Reference	
	Black/African American	0.8 (0.5, 1.3)	.4
	Asian	0.9 (0.3, 2.5)	.8
	Other race	0.5 (0.3, 1.0)	.06
**Hispanic ethnicity**		0.9 (0.5, 1.8)	.8
**Private insurance** ^b^		2.0 (1.3, 3.1)	.002
**Asthma severity** ^b^			
	Intermittent	Reference	
	Mild persistent	1.9 (1.2, 3.0)	.009
	Moderate/severe persistent	1.9 (1.0, 3.5)	.04
**On asthma controller medication**		2.0 (1.5, 2.7)	<.001

^a^This model also controlled for primary care practice—odds ratios are not displayed. Practice setting and region were entered into models but dropped due to collinearity.

^b^Data on insurance type and asthma severity were only available for PeRC patients (7120/9133 or 77.96% of the total)—the results presented for these variables are from models including only PeRC participants, whereas the results for all other variables are from models including all participants.

### Effect of Phone Versus Letter Recruitment 

Portal adopters reported how they learned about the portal. Letters to families resulted in the greatest number of enrolled families. Overall, 208 of 237 enrolled received a letter, 17 received a phone call, 35 heard about it from their child’s doctor, nurse practitioner, or nurse, and 3 from an informational card at the practice (25 reported multiple methods). Overall, 2.6% of children contacted by mail only enrolled, whereas 2.7% of those randomized to receive phone calls (they previously received letters) enrolled.

### Effect of Portal Use on Asthma Management

Those with uncontrolled asthma commonly planned changes in management after portal use. After completing the first survey, 16% reported an intention to change their child’s asthma medication, 27% to contact their child’s doctor, and 20% to make a change to their child’s environment, with more than one-third (27 parents, 36%) reporting an intention to take at least one action. On follow-up surveys, 22% reported a medication change, 41% reported contacting their child’s doctor, and 16% reported making a change to their child’s environment (Table 4).

Health records confirmed that portal completion was associated with changes in asthma care. Of the 76 children with uncontrolled asthma after the first survey, 20 (26%) had a medication change or refill within 30 days of survey completion, and 21 (28%) had an asthma-related primary care visit within 30 days (Table 5). These numbers represent a significant increase in medication changes or refills and asthma-related visits when compared with the same period the year prior for each child (14% increase in medication changes [95% CI, 2%, 27%] and 16% increase in visits [95% CI, 3%, 28%]). Results were similar in a sensitivity analysis that examined the 14-day period after portal use.

**Table 4:** Changes to asthma management planned and taken by families in response to receiving an uncontrolled result on the MyAsthma survey: based on parent survey.

		N (%)
**N children with uncontrolled asthma**		76
**Actions planned as of first survey (parent-reported)** ^a^		
	Contact doctor	20 (27)
	Change medications	12 (16)
	Change environment	15 (20)
**Actions taken as of second survey (parent reported)**		
	N uncontrolled with a follow-up survey completed	49
	Contacted doctor	20 (41)
	Changed medications	11 (22)
	Changed environment	8 (16)

^a^Parent/guardian reported being more likely or much more likely to take these actions after completing the MyAsthma survey

**Table 5:** Changes to asthma management planned and taken by families in response to receiving an uncontrolled result on the MyAsthma survey: based on electronic health record data

Actions taken, based on electronic health record data^a^	Within 30 days of survey completion, N (%) of children	In comparison period (the same 30-day period 1 year prior), N (%) of children	Difference between study year and previous year, N (%) of children (95% CI)
Medication change	20 (26)	9 (12)	+11 (+14% (2, 27))
Primary care asthma visit	21 (28)	9 (12)	+12 (+16% (3, 28))
Either action	30 (39)	14 (18)	+16 (+21% (7, 35))

^a^The denominator for all percentages from the electronic health record-based data is 76 (all children with an uncontrolled result on the first survey)

### Qualitative Results

Qualitative results revealed the importance of practice organization, family, and innovation characteristics to portal adoption (Table 6). Few health system factors were discussed and, when mentioned, clinicians disagreed about the value of incentives to promote portal adoption. Only 1 parent interviewed mentioned that an incentive (in this case from the research team) encouraged her to try the portal. For practices or clinicians, 3 primary themes emerged: the need for well-defined and coordinated workflows, the importance of practice responsiveness to portal surveys, and challenges related to identifying children with asthma through the EHR, which resulted in the recruitment of children without recent symptoms. In terms of workflow, clinicians and parents described that portal implementation was facilitated at practices that designated a specific person to coordinate the portal surveys and hampered when workflows were not well defined. Specifically, clinicians in 2 large urban practices reported being short staffed, lacking infrastructure in terms of care coordinators, and uncertainty about the ideal workflow for managing portal surveys. In addition, a perceived need among clinicians for more training diminished enthusiasm at some sites. Among those interviewed, parents of children with well-controlled asthma found MyAsthma less useful if they did enroll. Clinicians, especially in less affluent settings, perceived a lack of computer access as a barrier for parents. At the innovation level, features of MyAsthma that families and clinicians valued included facilitation of communication, increasing family awareness of and responsiveness to uncontrolled asthma, and ease of portal use.

**Table 6:** Qualitative results of interviews with 22 families and 10 focus groups with primary care clinicians.

Level	Theme	Specific barriers and facilitators and representative quotations
Structural/health system	Financial incentives	*Incentives paid to families may encourage use* (facilitator, 6 practices and 1 enrolled parent) “Incentive would grab [parent’s] attention. It sounds like [using the portal] doesn’t take a lot of time or require a lot of work.”—Clinician focus group *Incentives to families or providers would not encourage adoption* (barrier, 3 practices) “I don't really know that more money would incentivize…compliance.”—Clinician focus group
Practice/clinician	Workflow and coordination	*Coordination of portal surveys by a particular staff member facilitated implementation* (facilitator, 4 practices) “… we had a particular person who was spearheading it, so it wasn’t like 5 different people were picking up the faxes, they went to a central person and that person distributed it from there, and that I think was helpful. It would've been more confusing would we have had everybody in that.”—Clinician focus group *Lack of an established workflow* (barrier, 3 practices) “I also did not actively push [portal use] at all. I have fear of MyChart. That I’ll have not a good ability to manage the in-basket, and that our support team, while excellent, is already stretched, and not…we haven’t built a great infrastructure in terms of care coordinators being able to handle first line, so until we feel secure that’s in place and really well running, it feels like we are putting the cart before the horse.”—Clinician focus group *Lack of training for practices impeded effective use* (barrier, 6 practices) “Triage was not trained and did not know what questions were asked in the portal so found calling patients to follow up difficult.” —Clinician focus group
	Practice responsiveness to surveys	*Responsiveness by practices encourages use* (facilitator, 4 practices and 3 enrolled parents) “I had a mom that was really happy I called; that the office followed through, she was like I'm really glad you guys called me…it just felt good to type something in and get a response”—Clinician focus group “We always received a follow-up phone call from our pediatrician just making sure that we didn't have any questions, so I thought it was a great, you know, communication tactic”—Enrolled parent *Lack of follow-up by practices discouraged continued use * (barrier, 4 enrolled parents) “… I didn't really get any feedback or whatever from my doctor either way. So maybe… if it was actually hooked into responses from my doctor, then I would be more apt to use it...there was no interaction with my doctor's office or whatever, with doing that. I didn't really understand...does my doctor, does he see our answers and everything that goes into that.”—Enrolled parent
	Identification of children with asthma from the EHR	*Challenges selecting eligible patients using the EHR* (barrier, 2 practices) “…the selection process by which the patients were identified, needed, um, tweaking… It was too broad. It identified patients with a diagnosis of asthma in some cases quite a bit distant past. Or they might have had a diagnosis of wheezing per se, not, not asthma. …The family’s orientation was ‘my child doesn’t have asthma.’”
Parent/child	Asthma severity	*Parents of children with well-controlled asthma found less utility in the portal and were less likely to use it* (barrier, 3 practices, 3 enrolled parents, 3 unenrolled parents) “I noticed a lot of that was geared towards kids that are pretty severe, having multiple visits, stuff like that. We actually had a pretty mild winter here, so we really didn’t have a ton of asthma. We live in a pretty small rural area with pretty clean air so we just don’t see the severity that we used to see in [other areas].” —Clinician focus group
		“I guess for someone whose asthma is very well controlled like my son's, it is not really useful. If we were having difficulty then I guess it could have been better but we didn't really need it.”—Enrolled parent
		“My son's asthma is not very severe, so I think that if it was a significant daily type of problem for our family then I probably would have been interested in something like that, but we really don't have any trouble at all controlling his asthma. For us, at this point, it is really very simple for us to control. He every once in a while needs his inhaler, and that's about it“—Unenrolled parent
	Computer/Internet access	*Lack of computer/Internet access* (barrier, 5 practices) “…There might have been some access issues, we have, definitely a poorer population up here so not everybody has a computer, they might not want to access it on their phone.” —Clinician focus group
Innovation	Communication	*Portal use improved communication between families and primary care practices* (facilitator, 5 practices and 6 enrolled parents) ”I think functionally the portal was easy, it’s a way of patient physician communication to happen without the utilization of an office visit. It was a way of patients checking in saying, this is how its going and so that there’s better communication, optimizing the situation and cases where an alert was sent out where the patient really wasn’t doing that well. Well, we could move ahead and schedule them and find out why.“—Clinician focus group *“* It propelled me to call my doctor more…and to ask the appropriate questions.”—Enrolled parent

	Ease of portal sign up and use	*Portal was accessible and easy to use* (facilitator, 1 practice and 8 enrolled parents) “I think functionally the portal was very easy.”—Clinician focus group “It was extremely easy, especially for someone who is not the best on a computer, so it was very straightforward and asked appropriate questions, and easy, honestly, it really was an easy experience…”—Enrolled parent “Everything was, it's set up good, it's easy to get on to, if you have questions you wanna ask, I mean, it's simple, it's basic, and I am not a high tech person at all, I can barely use my iPhone without wanting to throw it across the room. So it was actually very easy, very easily accessible.”—Enrolled parent *Time burden involved in completing monthly surveys* (barrier, 1 enrolled and 2 unenrolled parents) I think when I had to report back to the doctor once a week/once a month, I think that's probably what made me, I don't feel like doing that.”—Unenrolled parent
	Portal increases family responsiveness to changes in asthma control	*Portal survey increased families’ awareness of and responsiveness to changes in asthma control* (facilitator, 2 practices and 7 enrolled parents) “At the beginning, I never would have thought that his asthma was uncontrolled, so that was helpful for me to see that his asthma was uncontrolled and now I have it controlled”—Enrolled parent “I think it alerted [parents] to signs and send them things to look for. I find that sometimes families are not in tune with what their child’s symptoms are. And this kind of alerted them to these are things you need to look for to see if your child is actually under good control. Because unless they are audibly wheezing or going through coughing fits, they wouldn’t see it otherwise.”—Clinician focus group “…After doing the survey seeing where my child was in terms of control, I felt like often times I thought it was well managed but it really wasn’t and there were things that I could discuss with the doctor and things that I could do to improve so he didn’t go to the emergency room and things like that.”—Enrolled parent “I may not have been consciously tracking her flares… for me it allowed me to look really in depth about how often was she really having a flare where I may not have been realizing it in the past, and to be better able to track how often her flares are and if she is really controlled”—Enrolled parent




## Discussion

### Principal Findings

We conducted a mixed-methods, multisite implementation study involving practices from 11 states to assess the feasibility for pediatric primary care practices of using a portal to facilitate communication between clinicians and families regarding asthma treatment, to assess the impact of portal use on asthma care for children with poorly controlled asthma, and to assess barriers and facilitators of portal adoption and sustained use. Overall, we found low rates of portal adoption and sustained use that varied from 0.6% to 13.6% across study practices. However, for those children with uncontrolled asthma, parent use of the portal was associated with a significant increase in asthma medication changes/refills and asthma visits to primary care practices. Qualitative methods underscored the importance of coordinated practice workflows, including practice responsiveness to portal surveys to implementation success. Parents, especially those with children with uncontrolled asthma, were motivated to continue using the portal because it facilitated a better understanding and tracking of asthma control.

Researchers and health systems in other settings have described low rates of portal adoption. For example, a study of the adoption of a portal for parents of children with cystic fibrosis, juvenile idiopathic arthritis, or diabetes reported that only 28% of invited families obtained a portal account, and only 48% of those (13% total) actually used the portal [23]. Even lower activation rates were observed in a study of a non–disease-specific portal in pediatric primary care, where rates of adoption have been lower than in adult health care settings [24]. Although prior studies found that patients with chronic diseases were more likely than others to register for or use a portal than others [25,26], the adoption rate in our population of children with asthma was quite low. Our qualitative results revealed that, at least in part, the low participation rate resulted from the inclusion of children that parents perceived had well-controlled asthma. These results are consistent with studies in diabetes that found that patients who believed their disease was well controlled felt that entering information over time was unnecessary [27] and were less likely to enroll [28].

Adoption may also have been limited by practices’ infrastructure and workflow for managing electronic receipt of patient-reported information. In our qualitative study, both clinicians and families highlighted the importance of coordinated and responsive workflows to implementation success. Workflow issues have been described previously as a challenge to portal implementation [5,27,29] and a reason for variability in adoption between practices [5,24]. In a case report of the portal adoption experience at 4 different adult primary care practices, practices with strong leadership and high staff engagement had higher rates of enrollment [5]. Learning collaboratives focused on workflow redesign in family medicine practices resulted in rates of portal use exceeding 25% of patients [26]. Especially relevant to the 32 billion dollar Federal Meaningful Use Program in the United States [30], these findings underscore the importance of integrating the portal into office systems and focusing provider and staff attention on their use.

The value to parents, practices, and the health system of implementing portals depends on their ability to improve communication and, ultimately, outcomes. Although adoption of the portal was low, portal use was associated with increased family and practice engagement in asthma management. These results are consistent with our prior pilot trial of MyAsthma, in which clinical outcomes including frequency of asthma flares and days of work missed by parents improved significantly among enrolled families [20]. Our finding that prescriptions and asthma visits increased among uncontrolled patients after using the portal is also consistent with studies among both children with autism and adults with diabetes that showed more active management after enrollment [31,32]. Mechanistically and as supported by our qualitative interviews, portal use may support disease management by improving patient–provider communication [33-36]. These findings support continuing effort to spur portal adoption and sustained use.

Collectively, the results of this study suggest multiple persistent barriers for the use of portals to support chronic disease management in pediatrics and that achieving high rates of adoption likely depends on the extent of existing practice infrastructure focused on disease management. We found that some families invited to participate did not consider their children to have active (or any) asthma. These results highlight the need to cautiously define the population to establish any metric for implementation success. In the case of asthma, researchers have developed definitions of an “asthma computable phenotype,” an algorithm based on data such as diagnosis, visits, medication, and laboratories, that accurately identifies patients with asthma [37,38]. Tailoring such definitions will be important for directing portals toward those most likely to benefit.

### Limitations

This study had several limitations. First, although we enrolled practices from 11 states and practices varied greatly in adoption, slightly more than half of practices were from a single health system, potentially limiting the generalizability of results. In addition, although the asthma portal was implemented within primary care practices, it was implemented within the context of a research study. Findings may not reflect the results that would be observed if practices implemented a portal themselves. Third, this study had a relatively short follow-up period, limiting our ability to assess sustained use over a longer timeframe. Fourth, in our analysis of changes to medication and visits among children with uncontrolled asthma, we were unable to adjust for asthma severity due to limited sample size. Finally, we focused on a single chronic condition; however, asthma is a common chronic condition for which clinical trial evidence supports improved outcomes with portal use [20].

### Conclusion

Despite the potential for real benefits to communication and child health outcomes, results of this multisite implementation study suggest that achieving high levels of portal adoption is unlikely in the short term. Many practices will require redesigned and coordinated workflows and will need to develop targeted outreach to families of children with poor asthma control to ultimately support the use of this technology.

## Abbreviations

CHOP: The Children’s Hospital of Philadelphia
EHR: Electronic health record
PeRC: Pediatric Research Consortium
PROS: Pediatric Research in Office Settings
